# Movements and Habitat-Use of Loggerhead Sea Turtles in the Northern Gulf of Mexico during the Reproductive Period

**DOI:** 10.1371/journal.pone.0066921

**Published:** 2013-07-03

**Authors:** Kristen M. Hart, Margaret M. Lamont, Autumn R. Sartain, Ikuko Fujisaki, Brail S. Stephens

**Affiliations:** 1 Southeast Ecological Science Center, U.S. Geological Survey, Davie, Florida, United States of America; 2 Southeast Ecological Science Center, U.S. Geological Survey, Gainesville, Florida, United States of America; 3 Scientific R&D, Support to U.S. Geological Survey Southeast Ecological Science Center, Cherokee Nation Technology Solutions, LLC, Davie, Florida, United States of America; 4 Ft. Lauderdale Research and Education Center, University of Florida, Davie, Florida, United States of America; 5 Department of Wildlife Ecology and Conservation, U.S. Geological Survey and Florida Cooperative Fish and Wildlife Research Unit, University of Florida, Gainesville, Florida, United States of America; Texas A&M University-Corpus Christi, United States of America

## Abstract

Nesting strategies and use of important in-water habitats for far-ranging marine turtles can be determined using satellite telemetry. Because of a lack of information on habitat-use by marine turtles in the northern Gulf of Mexico, we used satellite transmitters in 2010 through 2012 to track movements of 39 adult female breeding loggerhead turtles (*Caretta caretta*) tagged on nesting beaches at three sites in Florida and Alabama. During the nesting season, recaptured turtles emerged to nest 1 to 5 times, with mean distance between emergences of 27.5 km; however, several turtles nested on beaches separated by ∼250 km within a single season. Mean total distances traveled throughout inter-nesting periods for all turtles was 1422.0±930.8 km. In-water inter-nesting sites, delineated using 50% kernel density estimation (KDE), were located a mean distance of 33.0 km from land, in water with mean depth of −31.6 m; other in-water inter-nesting sites, delineated using minimum convex polygon (MCP) approach, were located a mean 13.8 km from land and in water with a mean depth of −15.8 m. Mean size of in-water inter-nesting habitats were 61.9 km^2^ (50% KDEs, n = 10) and 741.4 km^2^ (MCPs, n = 30); these areas overlapped significantly with trawling and oil and gas extraction activities. Abundance estimates for this nesting subpopulation may be inaccurate in light of how much spread there is between nests of the same individual. Further, our results also have consequences for critical habitat designations for northern Gulf loggerheads, as protection of one nesting beach would not encompass the entire range used by turtles during breeding seasons.

## Introduction

Nest site fidelity is a strategy used by many species, including marine turtles, to help increase reproductive success [1]–[4]. Marine turtles return from in-water foraging grounds to nest at the region of their birth, and within each nesting season they will deposit a clutch of eggs approximately every 9–20 days [1]. Schroeder et al. [5] and Miller [6] reported the typical distance between successive loggerhead nests as 5 km, and inter-nesting distances for several of the world’s most significant loggerhead nesting beaches were reported to be less than 10 km [7]–[9]. By exhibiting within and among season fidelity to their natal beach, marine turtles are depositing their eggs in an area that has proven itself capable of successfully incubating and hatching eggs. Development of satellite tracking technology has allowed an increase in knowledge of the in-water distribution and habitat use of sea turtles [10]–[15], however, these subjects are just recently being addressed in the Gulf of Mexico [16], [17] and few include the inter-nesting period (but see [18]). This lack of knowledge may have serious consequences; underestimating the size of inter-nesting habitat used by loggerheads means this threatened species may not be receiving the amount of protection necessary for population recovery.

With the increase in use of satellite tracking methods [13], [14], information on inter-nesting movements has grown. This research has revealed a dichotomy in loggerhead habitat-selection behavior during inter-nesting periods. Some turtles remained just offshore of the original nest site [19] whereas others made long-distance (>100 km) movements often into oceanic waters [20]–[22]. In addition, research suggests that in some locations loggerheads forage during the inter-nesting period, possibly in response to local conditions and resources; therefore, habitat use during the inter-nesting period may vary greatly among geographic locations [20], [23]–[26], [12]. Further, if turtles make longer distance movements away from the nesting beach during inter-nesting periods, the extent of inter-nesting habitat required in some areas may be much larger than the in-water area immediately surrounding nesting beaches (see Schroeder et al. [5]).

One of the largest nesting aggregations of loggerhead turtles in the Atlantic basin is found along the southeastern (SE) U.S. where about 80% of all nesting occurs and 90% of all hatchlings are produced [27]. Genetic studies have divided the Western Atlantic loggerhead nesting group into five subpopulations: 1. Northern (Florida/Georgia border to southern Virginia); 2. Peninsular Florida (Florida/Georgia border through Pinellas County, Florida); 3. Dry Tortugas (islands west of Key West, Florida); 4. Northern Gulf of Mexico (Franklin County, Florida through Texas); and 5. Greater Caribbean (all other nesting beaches throughout the Caribbean and Mexico) [28]–[30]. More recently, Shamblin et al. [31] completed a comprehensive genetic analysis that supported recognition of 10 management units for Northwest Atlantic loggerheads. Whereas estimated declines in nest abundance on the Atlantic and southwest (SW) coasts of Florida ranged from 29% to 37% between 1989 and 2006 [32], abundance of nests along the northern Gulf of Mexico declined by almost half from 1994 to 2010 [33]. Further, recent minimum population estimates (from 2001 to 2010) of adult female loggerheads within the northern Gulf of Mexico subpopulation ranged from only 323 to 634 individuals [34], the second smallest compared to the Dry Tortugas subpopulation (range of 258 to 496 individuals; [34]).

Globally, loggerhead turtle populations appear to be in decline [35], [36]. Witherington et al. [32] suggest that consistent interactions with commercial fisheries are the primary reason for this decline. In addition to direct mortality in trawling gear, studies have shown that shrimp trawling can damage benthic habitat and reduce invertebrate abundance [37]–[39] which may reduce loggerhead prey. The most recent Biological Opinion from NMFS [40] forecasts approximately 4,000 loggerhead deaths annually in US waters due to the shrimp trawling fishery. Most shrimp trawling occurs along the continental shelf in waters less than 18 meters deep during April through October which coincides with the inter-nesting habitat and seasonality for loggerhead turtles [41]. In addition, loggerheads in the inter-nesting habitat face other threats such as propeller injuries from sport-fishing vessels [42], habitat loss, degradation, and pollution [43]. In addition to these long-standing threats to loggerheads in the Gulf of Mexico, turtles nesting in this region faced a new and serious threat in 2010 when the Deepwater Horizon oil platform exploded resulting in the largest oil spill in US history [44], [45]. Such a regional incident could drive this relatively small Northern Gulf subpopulation of loggerheads [34] towards extinction. Understanding the movements, behavior, and habitat use patterns of these loggerheads is therefore necessary to adequately protect important upland and in-water habitats.

Little is known about nest site fidelity, movements, or locations of in-water inter-nesting habitat for loggerheads in the Northern Gulf of Mexico. Thus, our objectives in this study were to: (1) assess nest-site fidelity as well as timing of and distances between emergences; (2) describe loggerhead movements within the inter-nesting period; (3) spatially define in-water inter-nesting areas; (4) define characteristics of inter-nesting areas (i.e., bathymetry, distance from shore); and (5) assess overlap of inter-nesting areas with anthropogenic threats such as shrimp trawling and active oil and gas extraction activities.

## Materials and Methods

### Study Sites

**Figure 1:**
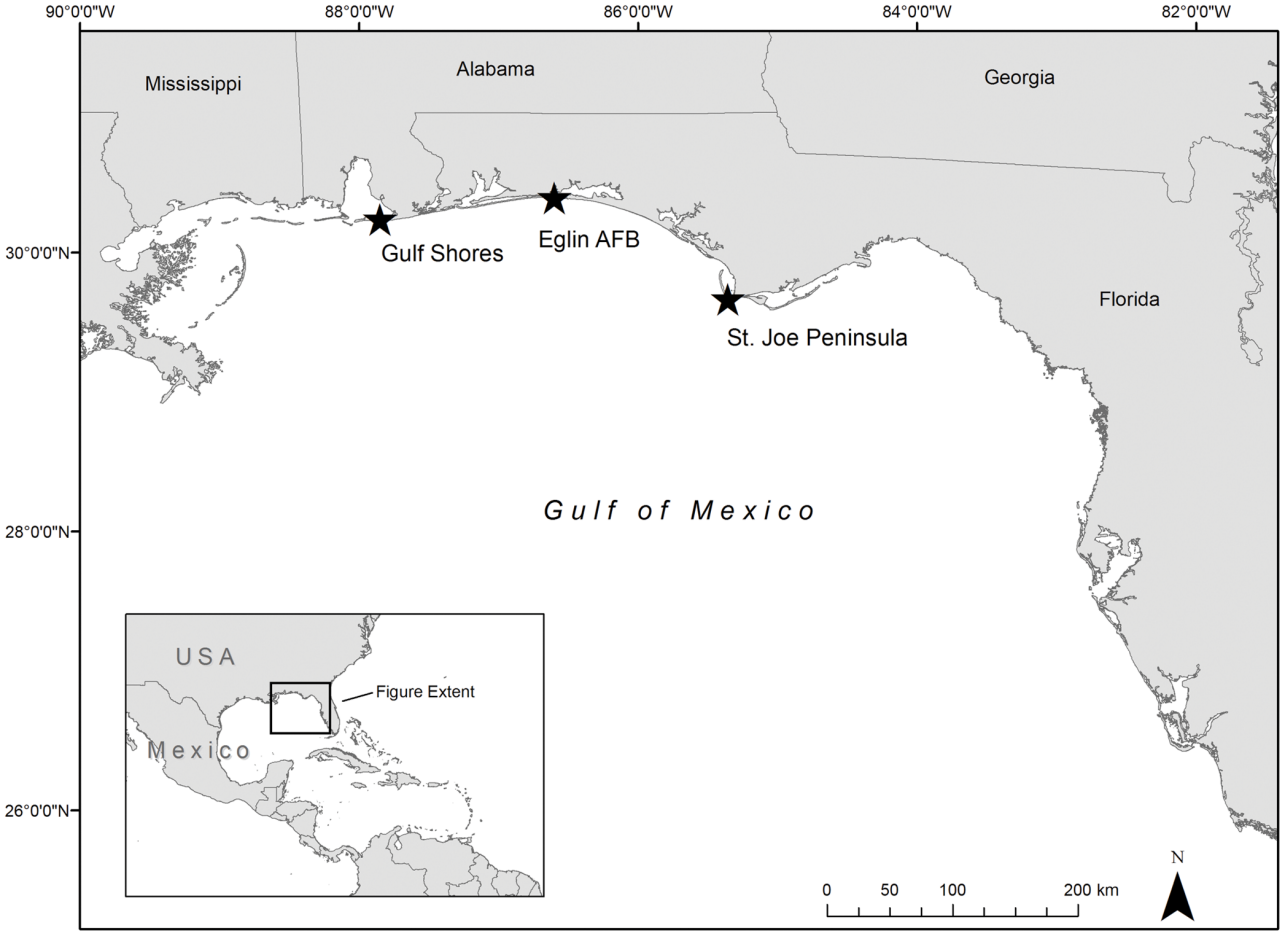
Study sites (stars) where adult female loggerheads (*Caretta caretta*) were intercepted, sampled, and satellite-tagged in 2010 (n = 4), 2011 (n = 13), and 2012 (n = 23).

Turtle tagging (see below) occurred at three study sites in the northern Gulf of Mexico (Figure 1). The Alabama (AL) site includes the Perdue Unit of the U.S. Fish and Wildlife Service Bon Secour National Wildlife Refuge and adjacent private lands in Baldwin County. The Florida sites comprise approximately 17 km of beach along the St. Joseph Peninsula (SJP) that includes 5 km of Eglin Air Force Base (EAFB) property and 12 km of public beach, and 18 km of beach owned by EAFB on Santa Rosa Island (Figure 1) in Northwest Florida (NW FL). These locations represent the eastern (SJP), middle (EAFB) and western (Alabama) extents of known loggerhead nesting in the northern Gulf of Mexico [27] and are separated by approximately 250 km (straight line distance).

### Turtle Capture and Transmitter Deployment

In the northern Gulf of Mexico, female loggerheads nest from approximately May 1 to September 1 [33]. In AL, nightly surveys were conducted from 9 pm to 6 am every day from 1 June to 30 June. On SJP, nightly surveys were conducted from 9 pm to 6 am every day from 15 May to 15 Aug. On EAFB, nightly surveys were conducted from 9 pm to 6 am for one week in July 2012 (10 to 17 July). Between 2010 and 2012, we intercepted and tagged 39 loggerheads after they nested; one turtle was captured and tagged twice in AL, in 2011 and 2012 for 40 captures (Table 1).

**Table 1:** Summary of satellite-tracking details for adult nesting loggerheads (*Caretta caretta*) in the Northern Gulf of Mexico, 2010–2012.

Tag Number	Size (CCL-tip, cm)	Tracking Period (days)^a^	Inter-nesting period (days)^b^	Encounters -nests and false crawls (mean inter-nesting interval in days)	Mean Distance (km) between observed emergence locations (number of distances)	Total distance moved (TDM) during Inter-nesting period (km)	TDM/day (km)
***Gulf Shores, Alabama***							
108170	95.2	6/6/2011–8/31/2011 (86)	ND	1	NA	NA	NA
106360	92.3	6/7/2011–8/31/2011 (85)	6/7/2011–8/4/2011 (58)	3 (22.5)	7.1 (2)	836.0	14.4
108172	93.9	6/8/2011–8/31/2011 (84)	6/8/2011–8/31/2011 (84)	3 (22)	129.0 (2)	2683.4	31.9
106345^c^	90.1	6/9/2011–8/31/2011 (83)	6/9/2011–8/31/2011 (83)	2 (42)*	3.5 (2)**	2163.4	26.1
108171	90.4	6/9/2011–8/31/2011 (83)	ND	1	NA	NA	NA
106337	93.6	6/11/2011–8/31/2011 (81)	6/11/2011–8/31/2011 (81)	3 (21.5)	8.7 (2)	1800.0	22.2
108173	95.9	6/11/2011–8/31/2011 (81)	ND	1	NA	NA	NA
108174	88.0	6/13/2011–7/11/11 (28)	ND	1	NA	NA	NA
106358	92.5	6/14/2011–8/31/2011 (78)	ND	1	NA	NA	NA
106361	92.0	6/15/2011–8/31/2011 (77)	ND	1	NA	NA	NA
108961	91.5	7/23/2011–8/31/2011 (39)	ND	1	NA	NA	NA
108964	94.6	7/30/2011–8/31/2011 (32)	ND	1	NA	NA	NA
108965	87.0	7/31/2011–8/31/2011 (31)	ND	1	NA	NA	NA
119940	94.2	6/1/2012–8/8/2012 (68)	6/1/2012–8/7/2012 (67)	3 (19.5)	13.0 (2)	2619.3	39.1
119941	92.0	6/3/2012–8/28/2012 (86)	6/3/2012–7/26/2012 (23)	2 (19)	118.7 (1)	2110.3	39.8
119943	97.5	6/4/2012–8/31/2012 (88)	6/4/2012–8/29/2012 (86)	1	NA	2230.4	25.9
119938	97.5	6/4/2012–8/31/2012 (88)	ND	1	NA	NA	NA
119924	95.4	6/5/2012–8/31/2012 (87)	6/6/2012–8/31/2012 (86)	2 (12)	7.8 (1)	2897.8	33.7
119944^c^	90.8	6/7/2012–8/31/2012 (85)	6/7/2012–8/29/2012 (83)	1	NA	1324.6	16.0
119946	95.0	6/9/2012–8/31/2012 (83)	6/9/2012–8/29/2012 (81)	2 (13)	254.6 (1)	1220.4	15.1
119945	98.9	6/9/2012–8/8/2012 (60)	none	1	NA	NA	NA
119947	98.5	6/13/2012–8/12/2012 (60)	6/13/2012–8/12/2012 (60)	1	NA	725.1	12.1
119923	95.6	6/13/2012–8/31/2012 (79)	6/13/2012–8/31/2012 (79)	2 (12)	12.9	2491.1	31.5
***St. Joe Peninsula, Florida***							
57656	99.5	7/26/2010–8/31/2010 (36)	ND	2 (24)	1.7 (1)	NA	NA
89971	103.2	7/27/2010–8/31/2010 (35)	ND	5 (14)	2.2 (4)	NA	NA
47755	97.2	8/3/2010–8/31/2010 (28)	ND	3 (12)	0.2 (2)	NA	NA
52968	90.0	8/4/2010–8/31/2010 (27)	ND	1	NA	NA	NA
53017	88.0	6/3/2012–6/29/2012 (26)	6/3/2012–6/26/2012 (23)	2 (26)	2.0 (1)	293.1	12.7
53016	102.0	6/4/2012–8/10/2012 (67)	6/4/2012–7/17/2012 (43)	4 (13)	4.4 (3)	515.4	12.0
53000	102.0	6/8/2012–8/27/2012 (80)	6/8/2012–8/27/2012 (80)	1	NA	1638.7	20.5
53164	102.0	6/8/2012–7/21/2012 (43)	6/8/2012–7/14/2012 (36)	5 (11.8)	2.4 (4)	220.8	6.1
119942	82.4	6/10/2012–8/31/2012 (82)	6/10/2012–8/31/2012 (82)	3 (21)	1.4 (2)	2221.0	27.1
119950	102.0	6/11/2012–8/6/2012 (56)	6/11/2012–8/5/2012 (55)	4 (17.3)	3.0 (3)	540.4	9.8
119949	100.0	6/11/2012–7/21/2012 (40)	6/11/2012–7/12/2012 (31)	4 (13)	1.7 (3)	399.4	12.9
119951	103.3	6/11/2012–8/2/2012 (52)	6/11/2012–7/18/2012 (37)	1	NA	422.0	11.4
119948	92.5	6/11/2012–8/31/2012 (81)	ND	5 (12.8)	1.7 (4)	NA	NA
119952a***	101.1	6/13/2012–7/6/2012 (23)	6/13/2012–7/6/2012 (23)	2 (23)	402.1 (1)	653.3	28.4
119952***	90.1	7/23/2012–8/31/2012 (39)	7/23/2012–8/29/2012 (37)	3 (11.5)	1.8 (2)	752.5	20.3
***Eglin AFB, Florida***							
120438	97.0	7/10/2012–8/31/2012 (52)	7/10/2012–8/31/2012 (52)	1	NA	2837.3	54.6
120439	102.5	7/11/2012–8/31/2012 (51)	7/11/2012–8/2/2012 (22)	2 (22)	54.8 (1)	531.7	24.2

a Tracking period = Either when transmitter stopped or until Aug 31 of tagging year (data analysis cut-off), whichever comes first.

b Inter-nesting period = as defined by SSM, from tagging date until the last 'inter-nesting point'.

c Same turtle tracked/observed in 2011 and 2012.

ND = not determined through switching SSM.

NA = Not applicable.

* Value only includes the interval (days) in 2011 between turtle's two nests.

** Value includes the distance between nests of 2011 and 2012.

*** Same transmitter used for tracking both turtles in 2012, in successive time periods.

Turtles were documented and outfitted with transmitters using established protocols [46]. Turtle interception and tagging followed methods similar to those in Girard et al. [16] and Hart et al. [18]. Briefly, we intercepted nesting loggerhead females after they had finished depositing their clutch on the beach. Immediately after marking each turtle with Inconel and PIT tags, we took standard carapace measurements, including curved (CCL) and straight (SCL) carapace lengths. We adhered platform transmitter terminals (PTTs) using slow-curing epoxy. We used three types of PTTs: SPOT5s from Wildlife Computers (Redmond, WA, USA; n = 8 in AL 2011; n = 3 in SJP 2010; n = 1 in SJP 2012; n = 2 on EAFB in 2012), SPOT5-MK10-AF tags from Wildlife Computers (n = 5 in AL 2011; n = 5 in SJP 2012) and Kiwisat 101s from Sirtrack (Havelock North, New Zealand; n = 1 in SJP 2010; n = 4 in SJP 2012). We streamlined attachment materials to minimize any buoyancy or drag effects on the turtle’s swimming ability and limited the epoxy footprint. Each tag was set to be active for 24 h d^1^.

### Sea Turtle Tracking

Location data were filtered using Satellite Tracking and Analysis Tool (STAT; [47]) available on www.seaturtle.org. Location classes (LC) 3, 2, 1, 0, A, and B were used to reconstruct routes and calculate straight-line and total distances that the turtles traveled. Locations were rejected if they were LC Z (for which no error estimation was available). Argos assigns accuracy estimates of <250 m for LC 3, 250 to <500 m for LC 2, 500 to <1500 m for LC 1, and >1500 m for LC 0 [48]. The estimated accuracy is unknown for LCs A and B, and locations failing the Argos plausibility tests are tagged as class LC Z. Both traditional least-squares location processing (2010) as well as Kalman-filtering (initiated in 2011 and applied in 2011 and 2012; [49]) of location data was performed by Argos. This newly-implemented Kalman-filtering algorithm provides more estimated positions and significantly improves position accuracy, most significantly for locations obtained in LCs A and B [50].

### Switching State-space Modeling

We used switching state-space modeling (SSM; [51], [52]) to characterize the movements of adult nesting loggerhead females in the Gulf of Mexico. The model was described in 2005 [53] and has previously been applied to model movement of marine animals including marine mammals (blue whales [54]), and turtles [52], [55]–[61], [17].

Argos satellite locations are recorded at irregular time intervals and are often less precise than published estimates [62] which can be misleading in making inferences even after ad-hoc filtering of outliers [55]. Switching SSM is recommended as the best analytical technique for enhancing Argos tracking data once post processed by removing land points and adding back in good Argos locations [63]. Switching SSM has two components accounting for location errors (observation error) and animal behavior [55], [58]; the observation error is based on the location quality class associated with Argos data. The two-state switching correlated random walk models the movement process which transits between two behavioral states (see Jonsen et al. [53] for more detailed model description and Eckert et al. [64] for equations). Earlier applications defined binary behavioral modes as ‘foraging’ and ‘migration’ (e.g., Breed et al. [58]); however, since we tagged turtles during nesting seasons, we defined the behavioral modes as ‘foraging and/or nesting’ and ‘migration’. The observation equation translates observed locations to true unobserved locations at equal time intervals.

We specifically used SSM to estimate the time period that each satellite-tagged loggerhead was in inter-nesting and the location of inter-nesting sites. We applied a model used in Breed et al. [58], which is a modified version of a model described in Jonsen et al. [53] that estimates model parameters by Markov Chain Monte Carlo (MCMC) using WinBUGS via the software program R. We used all tracking data except for LC Z, and we fit the model to tracks of each individual turtle to estimate location and behavioral model every eight hours from two independent and parallel chains of MCMC. Our samples from the posterior distribution were based on 10,000 iterations after a burn-in of 7,000 and thinned by five. The convergence was monitored by observing model parameters of two independent chains that were mixed in the trace plots as suggested by Breed et al. [58].

### Nest Site Characteristics and Nest-site Fidelity

During the inter-nesting period, we encountered many turtles on land more than once. For these turtles, we calculated 1) distances between successive emergences, and 2) mean length of inter-nesting intervals (in days). We classified whether a turtle displayed nest-site fidelity based on the distance between successive emergences (≤5 km from previous nest = nest-site fidelity; >5 km from previous nest = no nest-site fidelity; [5]). We determined whether turtle size affected nest site fidelity. In addition, using filtered satellite location data, we calculated the total distance moved by each turtle in the inter-nesting period (from capture date to the last inter-nesting location as defined by SSM) by adding up the straight-line distances between successive points; we determined if this distance correlated with distance between successive nest sites.

### In-water Inter-nesting Areas

Using a switching SSM allowed us to interpret fine-scale behavioral information within the turtle tracks. After fitting the switching SSM to individual loggerhead tracks, we identified locations where turtles were in inter-nesting mode. From these inter-nesting periods, we filtered out locations deeper than 200 m (neritic zone cutoff) along with any other obviously erroneous locations (on land, spatially very distant, etc.); Hawkes et al. [15] found that adult female loggerheads in the SE US did not generally leave the waters of the continental shelf (<200 m). If an individual inter-nesting period was at least 20 days in length, we also generated mean daily locations to minimize autocorrelation using the filtered locations within the foraging area; the resulting coordinates provided raw data for kernel density estimation (KDE). Kernel density is a non-parametric method used to identify one or more areas of disproportionately heavy use (i.e. core areas) within a home-range boundary [65]–[67], with appropriate weighting of outlying observations. We used the Home-Range Tools for ArcGIS extension [68] and fixed-kernel least-squares cross-validation smoothing factor (h*_cv_*) for each KDE [69], [70]. When we observed unequal variance of the *x* and *y* coordinates, we rescaled the data to select the best bandwidth (following [70], [71]). We used ArcGIS 9.3 [72] to calculate the in-water area (km^2^) within each kernel density contour (50% and 95%) and to plot the data; we used 95% KDEs to represent the overall home foraging range, and the 50% KDEs to represent core area of activity at foraging sites [73]. For turtles with multiple inter-nesting periods, we calculated a KDE at each inter-nesting period with at least 20 mean daily locations. We summarized data for inter-nesting periods until the transmitters stopped sending information or until 31 August of the reproductive year.

We also tested location data for and quantified fidelity to in-water inter-nesting locations using the Animal Movement Analysis Extension for ArcView 3.2. Using Monte Carlo Random Walk simulations (100 replicates), we tested tracks during the inter-nesting period for spatial randomness against randomly generated walks [73]. We bounded the range for random walks from 200 m –0 m bathymetry to include only the realistic extent of the in-water habitat for our animals during the study period. Tracks exhibiting site-fidelity indicate movements that are more spatially constrained rather than randomly dispersed [73]. In our analyses, coordinates were standardized due to unequal standard deviation of latitude and longitude for some animals.

To further characterize at-sea inter-nesting areas selected by individual loggerheads, we calculated the centroid of each turtle’s 50% KDE; if a 50% KDE included multiple activity centers, we calculated the centroid of the largest activity center. For inter-nesting periods (as defined by SSM) without 20 mean daily locations, we performed minimum convex polygon (MCP) analysis (100% of points; [74], [75]) using ArcMap 9.3 [72]. We then calculated the centroid of these MCPs.

### Inter-nesting Area Characteristics

We summarized the spatial configuration of inter-nesting centroids by calculating distances between and among centroids (both KDE and MCP derived) at each inter-nesting site, and the distance from each centroid to both the nearest land and the mainland. We also extracted depths for all points remaining after filtering (i.e., those passing a 5 km/hr swim speed limit, those not on land) within inter-nesting periods. For turtles without a successful SSM run, we used filtered locations from the capture date until August 31 of the reproductive year. For those with a successful SSM run, we used filtered locations from the capture date until the last point in inter-nesting mode, including any migration points in between. For bathymetry, we used the NOAA National Geophysical Data Center (GEODAS) ETOPO1, 1 arc-minute global relief model of Earth’s surface (http://www.ngdc.noaa.gov/m,gg/geodas/geodas.html; accessed 26 January 2012).

To depict all inter-nesting locations used by turtles over time, we calculated the number of turtle-tracking days in grid cells (10 × 10 km); the grid extended across the extent of the Gulf of Mexico within the 200 m isobath. For the 27 turtle tracks we ran in SSM, we counted number of days each turtle was observed (turtle days) in each grid cell using all satellite locations except for LC Z during inter-nesting periods. For the 18 turtle tracks that did not run in SSM, we counted turtle days using all satellite locations except for LC Z. To explore likely correlates of inter-nesting habitat selection, we also calculated mean values for distances from the centroid of each grid cell center to the mainland, distance to mean tagging/release locations and bathymetry at the centroid of each grid cell. We used a generalized linear model (GLM) with log-transformation to analyze the effect of bathymetry and distance to the tagging location on turtle days spent during inter-nesting in each grid cell using SAS 9.1 GENMOD procedure. For all statistical comparisons, we used an alpha level of 0.05.

### Potential Overlap with Anthropogenic Activities

Finally, to help provide guidance for conservation and management actions with inter-nesting habitat, we also mapped the overlap of commercial trawling during May-August 2011 (time of inter-nesting) and the locations of active oil and gas platforms. We created a layer in ArcGIS 9.3 [72] using shrimp trawling data and statistical zone cutoffs provided by NOAA (Jim Nance, Amanda Frick, pers comm.). The layer for oil and gas platforms was obtained from http://www.data.boem.gov/homepg/data_center/mapping/geographic_mapping.asp, accessed on 8 November 2012; platforms with a past removal date were filtered out of the layer before mapping. We described these potential threats for each centroid in two ways: we provided the number of shrimp trawling days associated with the area containing the centroid, and we totaled the number of active oil and gas platforms within a 10 km buffer of each centroid.

## Results

### Turtles

Turtles (n = 39 individuals, n = 40 tracks) ranged in size from 82.4 to 103.3 cm curved carapace length (CCL; mean = 95.2±5.1 cm; Table 1). In a total of 2470 tracking days during inter-nesting, individual turtle tracking durations ranged from 23 to 88 d (mean ±1SD = 61.8±23.0 d; Table 1).

### Nest site Characteristics and Nest-site Fidelity

Loggerheads were encountered on the beach either nesting or false-crawling from 1 to 5 times each (mean±SD = 2.1±1.3 nests); 22 turtles were encountered nesting on more than one occasion (Table S1). Of these 22 turtles, the mean interval between encounters was 16.8±7.0 d (range 11 to 42 d). The mean straight-line distance between encounters ranged from 0.11–402.1 km (n = 45 emergences; mean±SD = 27.5±79.3 km; Table 1). We saw no relationship between turtle size and nest site fidelity (r = 0.116; p = 0.65) and no correlation between distance moved during the inter-nesting period and distance between subsequent nesting sites (r = −0.060; p = 0.81).

**Figure 2:**
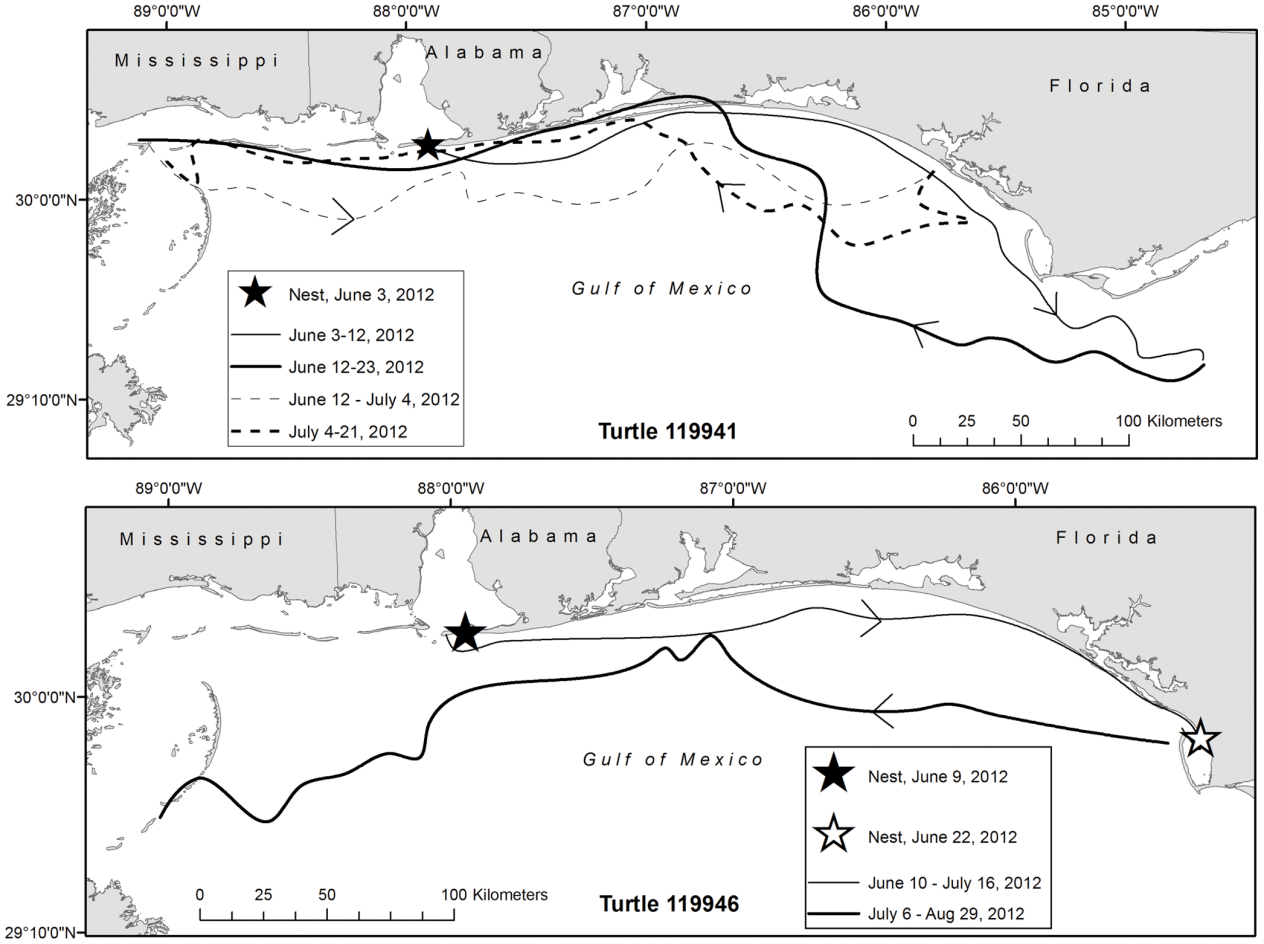
Loggerhead “exchanges” between study sites. Tracks of satellite-tagged adult female loggerheads (*Caretta caretta*) 119941 and 119946 during the inter-nesting period in 2012 (A); tracks of satellite-tagged adult female loggerheads 108172 (2011) and 119940 (2012) during the inter-nesting period (B).

A subset of these satellite-tracked turtles have been observed emerging in various sites throughout the Northern Gulf during a single nesting season (n = 5). These included turtle 108172 that nested on 8 June 2011 in AL and then on SJP in FL on 22 July 2011 (256.3 km away); turtle 119946 that nested 9 June 2012 in AL and then again on 22 June 2012 on SJP (254.6 km away); turtle 120439 that nested 11 July 2012 at EAFB and then 2 August 2012 at Fort Pickens, FL (54.8 km away); turtle 119941 that nested 2 June 2012 in AL and then false-crawled at Long Beach, MS on 22 June 2012 (118.7 km away); and turtle 119952a that nested on SJP on 13 June 2012 and then false-crawled on 7 July 2012 on Casey Key, FL (402.1 km away; see Figures 2a and 2b). Two other satellite-tagged turtles were observed traveling between the two main tagging sites (AL and SJP, FL) both within and among years. Turtle 108173 (RRA088; satellite- and flipper-tagged) originally nested on SJP on 27 June 2002 (MML, pers. observ.), was then observed nesting in Alabama on 11 June 2011, and then nested again on SJP on 6 July 2011. Turtle 119940 (RRN111; satellite- and flipper-tagged) was originally tagged nesting on SJP on 8 June 2006 (MML, pers. observ.) and was then observed nesting in AL on 1 June 2012 (see Figure 2b for 119940).

### In-Water Inter-nesting Areas

**Figure 3:**
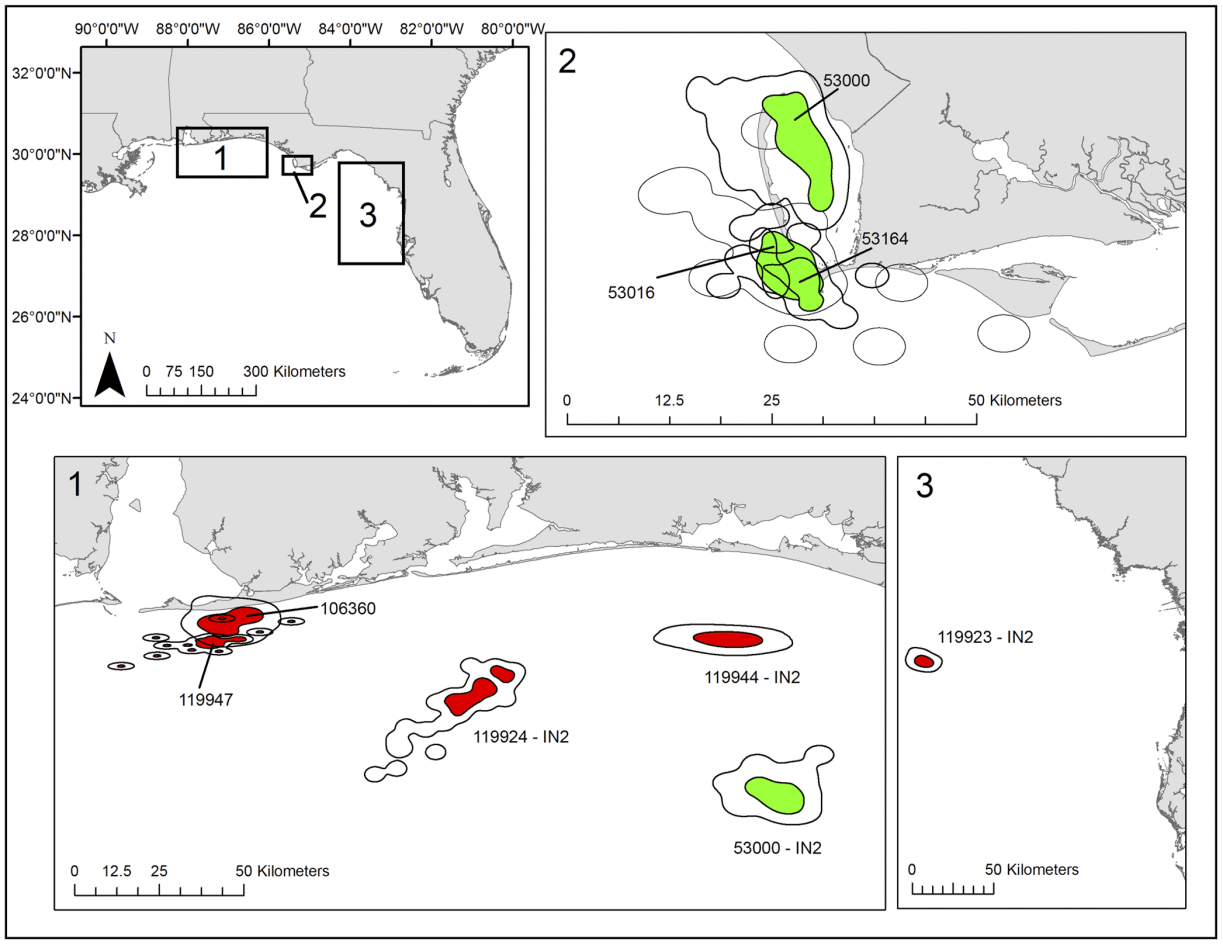
Plot of inter-nesting 95% (outer line) and 50% (shaded in) kernel density estimates (KDE) for adult Northern Gulf loggerheads (*Caretta caretta*) during inter-nesting. Red indicates turtles that were tagged at the AL study site and green indicates turtles tagged at the SJP study site.

We obtained SSM results for 25 turtles (Figure S1 and Table S2 provide two example SSM prediction paths and associated model parameters for turtles tagged in AL [119946] and SJP [53000]). Of these, 24 turtles had time periods classified as inter-nesting and other periods classified as migration (defined as consistent, directed movements; one turtle was only in migration mode for the duration of the study period). Eleven turtles showed only one inter-nesting period, whereas 13 turtles were in inter-nesting mode more than once throughout the study period. Overall inter-nesting periods as defined by SSM for these 24 turtles (from capture date to last inter-nesting point regardless of migration periods) totaled 1392 days and ranged from 22 to 86 d (mean±SD = 58.0±24.2 d; Table 1). Thirteen turtles (16 inter-nesting periods) had enough mean daily locations during an inter-nesting period for KDE analysis, but only 9 of 13 turtles (69%) displayed fidelity to at least one of these in-water locations (Table 2, Table S3). Therefore, we calculated KDEs for these 9, resulting in 10 KDEs (one turtle had two KDEs for two separate inter-nesting periods; Figure 3). During inter-nesting, we obtained 301 total mean daily locations for analysis; the overall size of 50% KDE core-use areas ranged from 18.1 to108.2 km^2^ (mean ±1 SD = 61.9±28.2 km^2^; Table 2). Because not every tracking day provided a turtle location, the time period during which turtles were resident at these sites differed slightly from the number of mean daily locations. Overall, turtles occupied 50% KDEs for a total period of 431 d (range 20 to 62 d; mean ±1 SD = 43.1±14.1 d; Table 2). We also calculated 40 total MCPs; 10 were for turtles with an existing KDE for the same period of time. For the 30 remaining MCPs, turtles occupied MCPs from 2 to 55 d (mean ±1 SD = 20.5±12.8 d) and the size of MCPs ranged from 3.0 to 3274.2 km^2^ (mean ±1 SD = 741.4±750.2 km^2^; Figure S2, Table S4).

**Table 2:** Kernel density estimation (KDE) results for loggerhead turtles (*Caretta caretta*) with successful state-space model (SSM) runs.

Tag Number	Inter-nesting dates for KDE (days)	KDE Bandwidth	Site Fidelity Test^a^	50% KDE area(km^2^)^b^	95% KDE area (km^2^)^b^	Centroid Depth (m)	Centroid to shore (km)
106360	6/7/2011–8/4/2011 (58)	0.64	Pass	86.6	257.2	11.0	3.8
53016	6/5/2012–7/17/2012 (42)	0.24	Pass	35.5	287.6	9.0	1.2
53000*	6/8/2012–7/24/2012 (46)	0.31	Pass	50.6	225.0	10.0	3.8
53164	6/8/2012–7/14/2012 (36)	0.3	Pass	18.1	107.9	5.0	0.9
119947	6/13/2012–8/12/2012 (60)	0.17	Pass	36.5	157.8	13.0	9.3
53000*	7/28/2012–8/27/2012 (30)	0.53	Pass	108.2	465.7	95.0	67.1
108172	8/11/2011–8/31/2011 (20)	0.25	Pass	76.7	354.6	67.0	68.0
119923	7/31/2012–8/31/2012 (31)	0.83	Pass	54.1	217.7	37.0	113.6
119924	7/16/2012–8/31/2012 (46)	0.18	Pass	88.7	432.6	37.0	36.2
119944	6/28/2012–8/29/2012 (62)	0.94	Pass	63.5	250.1	32.0	25.9
			*mean*	*61.9*	*275.6*	*31.6*	*33.0*
			*SD*	*28.2*	*113.6*	*29.4*	*38.4*

a All p values for site fidelity test were >99.0099.

b These values include only in-water area; any land within KDE contour was removed from total area.

* This individual had two areas suitable for KDEs within the same inter-nesting season. The two time periods were separated by a short 'migration' time of three days.

### Spatial Configuration of Inter-nesting Sites

Distances to the nearest land from 50% KDE centroids (n = 12) ranged from 0.9 to113.6 km (mean±SD = 33.0±38.4 km; Table 2). Bathymetry values (i.e., a proxy for water depths) at these centroid locations ranged from −95.0 to −5.0 m (mean±SD = −31.6± −29.4; Table 2). Distances to the nearest land from centroids of the 30 MCPs (for time periods with no KDE) ranged from 0 to 94.0 km (mean±SD = 13.8±18.9; Figure S2, Table S4). Bathymetry values at these locations ranged from −43.0 to 0.0 m (mean±SD = −15.8±11.2; one centroid location occurred on land).

### Total Distance Moved During Inter-nesting

**Figure 4:**
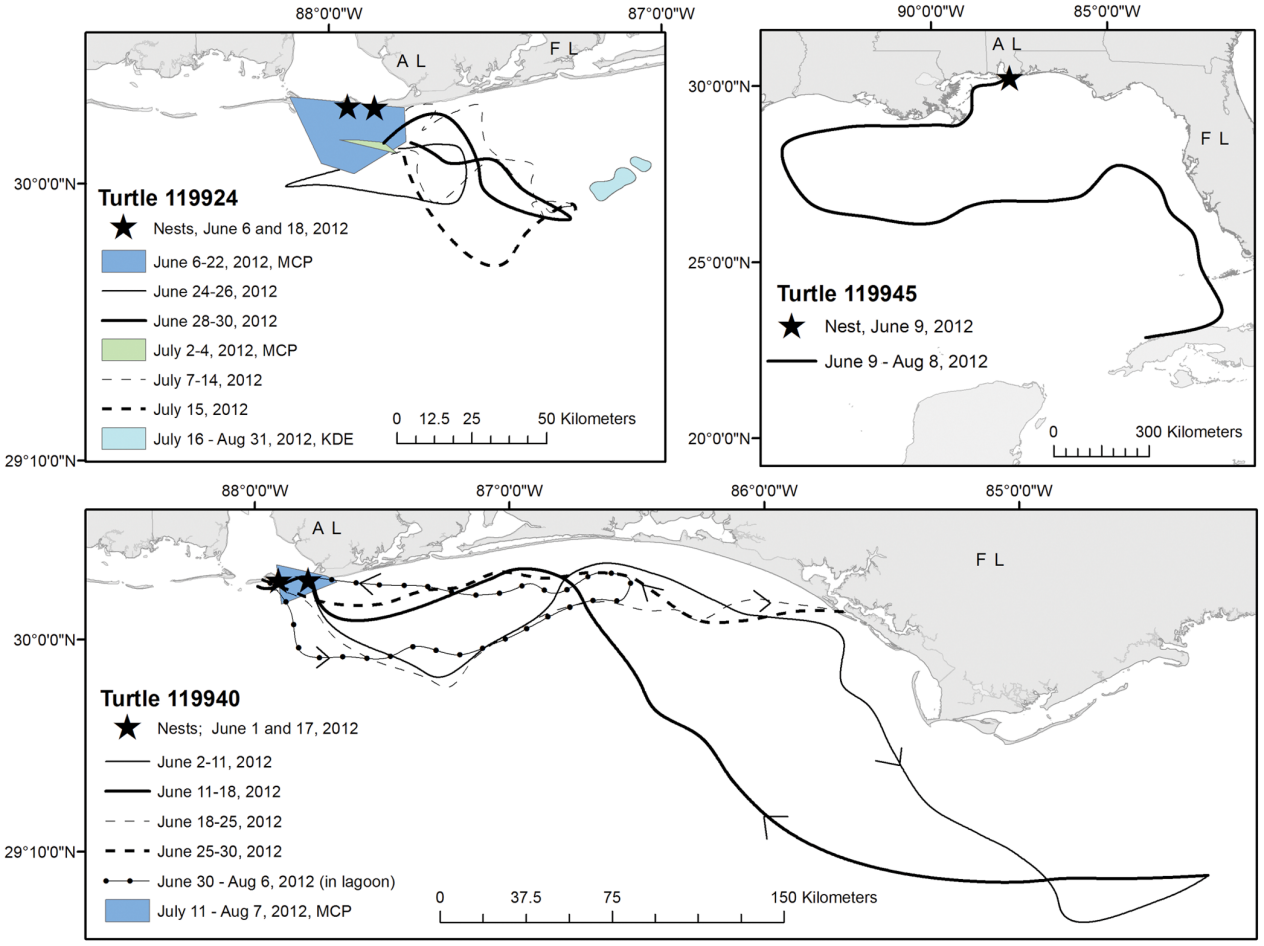
Examples of 3 different types of behavior for loggerhead sea turtles (*Caretta caretta*) in the Northern Gulf of Mexico, USA, during the inter-nesting period. Turtle 119924 displayed site-fidelity and selected a discrete habitat during inter-nesting periods; turtle 119945 wandered during inter-nesting, and did not return to land after nesting on 9 June 2012; turtle 119940 displayed nest-site fidelity but not site-fidelity to any in-water habitat during inter-nesting.

Total distances between successive inter-nesting in-water habitats (either MCP or KDE centroids) for the same turtle (n = 11) ranged from 78.6 to 726.8 km (mean±SD = 304.3±180.6 km; Table 3). For turtles with predicted inter-nesting times (n = 24), their total distances traveled ranged from 220.8 to 2897.8 km (mean±SD = 1422.0±930.8 km; Table 1). Corrected for time (total distance divided by number of days in the inter-nesting period), turtles traveled from 6.1 to 54.6 km/day (mean±SD = 22.8±11.6 km/day; Table 1). Movements during inter-nesting varied by turtle: some remained in neritic habitat near the nesting beach, some made long-distance movements but stayed in neritic habitat, and some used oceanic habitat during inter-nesting (Figure 4).

**Table 3:** Distances between successive minimum convex polygon (MCP) and kernel density estimate (KDE) centroids for loggerhead turtles (*Caretta caretta*).

			Distances between centroids			
Tag Number	Inter-nesting period (days)	No. of centroids	Centroid 1–2 (km)	Centroid 2–3 (km)	Centroid 3–4 (km)	Total (km)
***Gulf Shores, Alabama***						
108172	6/8/2011–8/31/2011 (84)	2*	726.8	NA	NA	726.8***
106345	6/9/2011–8/31/2011 (83)	3	31.0	206.2	NA	237.2
106337	6/11/2011–8/31/2011 (81)	4	31.5	20.2	196.60	248.3
119943	6/4/2012–8/29/2012 (86)	3	82.6	355.0	NA	437.6
119924	6/6/2012–8/31/2012 (86)	3	7.7	70.9	NA	78.6
119944	6/7/2012–8/29/2012 (83)	2*	236.9	NA	NA	236.9
119946	6/9/2012–8/29/2012 (81)	2*	345.0	NA	NA	345.0
119923	6/13/2012–8/31/2012 (79)	2*	394.1	NA	NA	394.1
***St. Joe Peninsula, Florida***						
53000	6/8/2012–8/27/2012 (80)	2**	94.0	NA	NA	94.0
119942	6/10/2012–8/31/2012 (82)	2	348.8	NA	NA	348.8
119952	7/23/2012–8/29/2012 (37)	2	199.8	NA	NA	199.8
					*mean*	*304.3*
					*SD*	*180.6*

* mixed MCP and KDE centroids.

** KDE centroids only.

*** Potential foraging area included in this total because turtle may have arrived at foraging ground before 8/31/11 cutoff date.

NA = not applicable.

### Water Depth used During Inter-nesting

**Figure 5:**
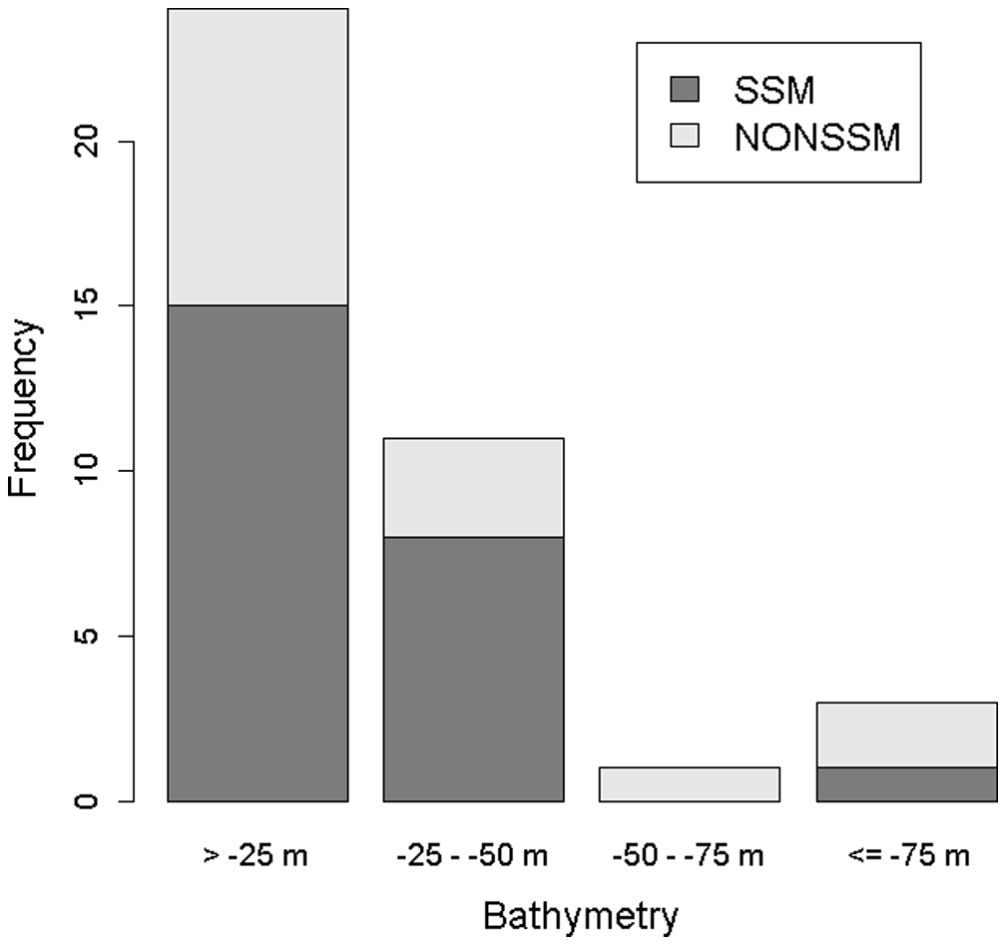
Bathymetry histogram for locations used by satellite-tagged loggerheads (*Caretta caretta*; n = 39 individuals) in the Northern Gulf of Mexico, 2011–2012. SSM = turtles for which state-space modeling was successful (n = 24); NONSSM = turtles for which SSM was not possible (n = 18).

We extracted 9,536 separate depth locations for all turtles during inter-nesting. The majority of locations (89.6%, 8,541 points) were in waters −50 m or shallower. The remaining 7.5% of locations (716 points) were in water −51 to −100 m deep, with 1% (98 points) in water −101 m to −150 m deep, 0.2% (19 points) in water −151 m to −200 m deep, and 1.7% (162 points) in water deeper than −200 m (Figure 5; Table S5). Size of turtles (CCL-tip) and mean bathymetry values per centroid were not significantly correlated (r = −0.174, p = 0.283). Mean centroid depths were 31.6 m (SD 29.4; Table 2) for KDEs and ranged from −1 m to −43 m for MCPs (Table S4).

### Turtle Inter-nesting Days per Grid Cells

**Figure 6:**
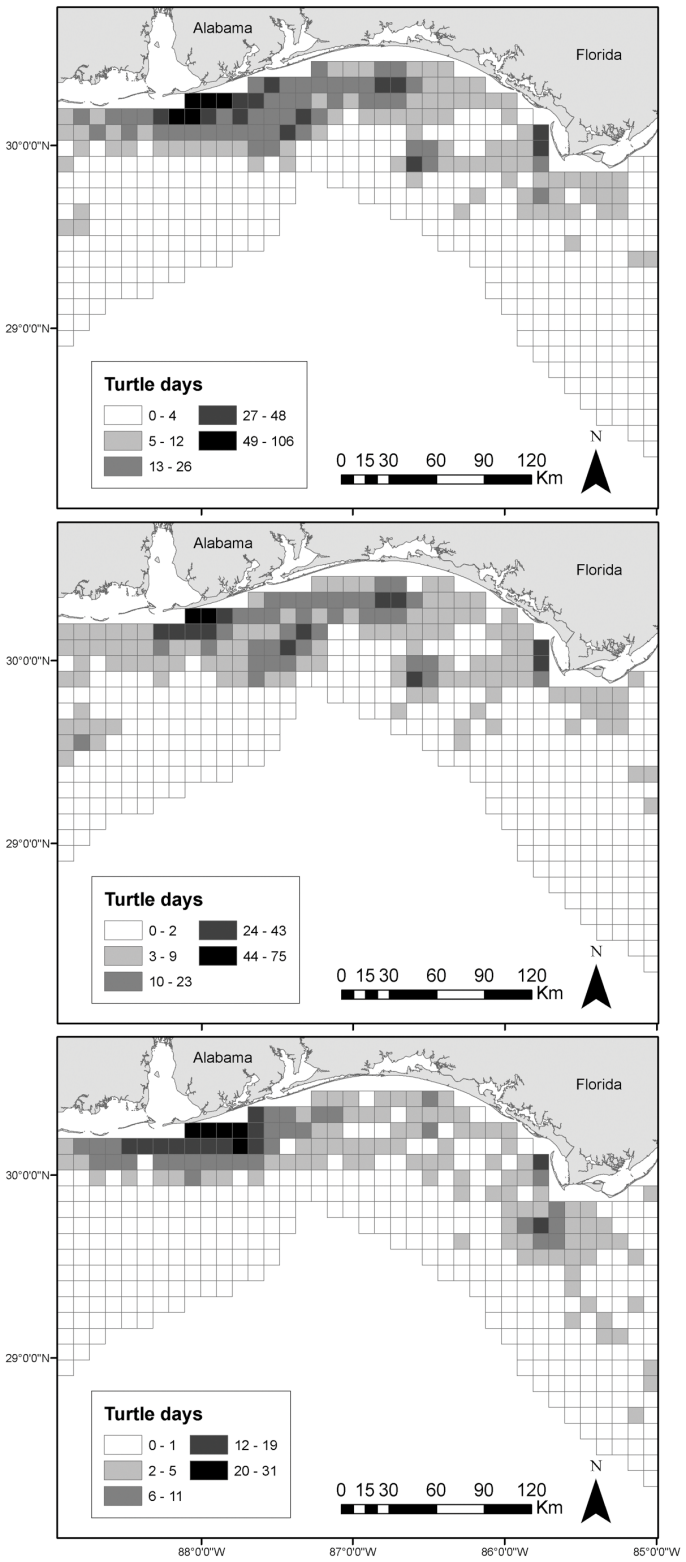
Number of loggerhead turtle (*Caretta caretta*) days spent for all (top), state-space modeled (SSM; middle) and non-SSM (bottom) turtles. Increasingly darker grid cells indicate a higher number of turtle-days per grid cell.

High numbers of turtle-days per grid cell occurred during inter-nesting in locations adjacent to nesting beaches. Bathymetry was a significant predictor of turtle days spent in each grid cell during inter-nesting (Chi-square = 797.56, *p<0.0001*; Figure 6); turtles spent longer periods in shallower grid cells.

### Potential Overlap with Anthropogenic Activities

**Figure 7:**
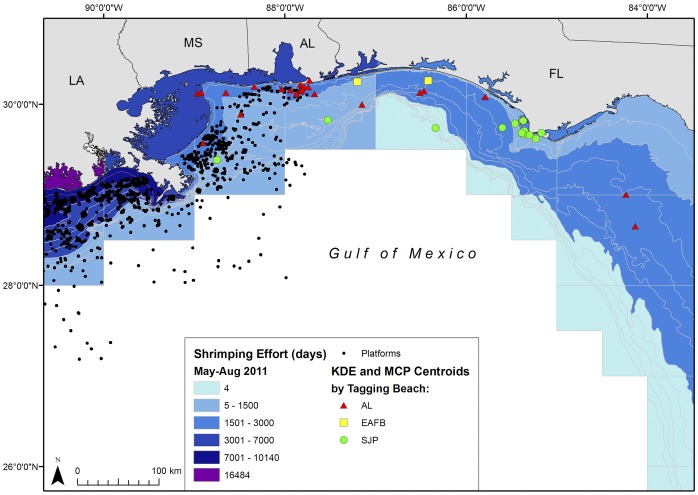
Centroid locations for inter-nesting habitat used by loggerheads (*Caretta caretta*) in the Northern Gulf of Mexico; kernel density estimates (KDE) and minimum convex polygon (MCP) centroids are shown in relation to active oil and gas platforms (black dots; data from www.data.boem.gov), and shrimp trawling effort (days; data provided by NOAA) from May to August 2011. Turtle tagging locations are distinguished by symbols as in legend.

Inter-nesting in-water habitat overlapped with trawled areas in all cases. Of 40 centroid locations representing in-water inter-nesting habitat, 60% occurred in areas with 1501–3000 days of reported trawling from May-Aug 2011; 27.5% occurred in areas with 5–1500 days, 7.5% occurred in areas with 3001–7000 days, and 5% occurred in areas with 4 days of trawling during this same time period (Figure 7, Table S6). Additionally, 35% of centroids were within 10 km of oil and gas platforms. Of these turtles, the numbers of platforms within 10 km ranged from 1 to 26 (mean±SD = 8.6±6.7 platforms; Table S6).

## Discussion

Our results demonstrate that female loggerhead turtles in the Northern Gulf of Mexico subpopulation express significantly less nest-site fidelity and make larger movements during the inter-nesting period than previously reported for this species [5], [19], even though they nest at approximately 2-week intervals like other loggerheads. We documented individual turtle use of both AL and SJP study sites within a single season; many Northern Gulf loggerheads may be using geographically separate nesting beaches regularly within one reproductive season. Further, in-water inter-nesting habitats were located in relatively shallow water in the Northern Gulf of Mexico, but habitats were not necessarily situated adjacent to nesting beaches. Such long-distance movements during inter-nesting may be typical for this subpopulation. These sites also overlapped considerably with locations of trawling and active oil and gas extraction activities.

### Nest site Characteristics and Nest-site Fidelity

While nest-site fidelity can vary among individual loggerheads, it has generally been accepted that females typically re-nest within 5-km of their original nest site and the mean distance between successive nest sites is similar among loggerhead populations [5]. Data from the east coast of Florida [8], [76], South Africa [7], and Australia [9] document mean distance between nest sites as <10 km. However, our findings suggest this may not be the case for the northern Gulf of Mexico subpopulation as we found the mean distance between nest sites was 27.5 km, and 18 turtles nested >5 km from their original nest sites. Recently, flipper-tag returns from Atlantic loggerheads (Wassaw Island, GA) on the East coast of the United States have shown a similar lack of nest-site fidelity within and across nesting seasons [77].

Although it has been suggested that neophyte nesters (presumably smaller turtles) may express lower nest-site fidelity than remigrants (presumably larger turtles; [78]–[80]), we saw no relationship between turtle size and nest-site fidelity for loggerheads in the northern Gulf, suggesting the age of turtles nesting in this region is not the reason for low nest-site fidelity. These findings have significant implications; if adult females display low nest-site fidelity and deposit clutches at multiple beaches within a nesting season, the number of nesting females in the region may be lower than estimated.

The SJP site supports some of the greatest density of nesting in this area [33], however, many turtles nesting on this beach will also nest on other low-density beaches in NW FL or AL. Therefore, critical habitat designations for loggerheads in the northern Gulf of Mexico must not be limited to high density nesting beaches. Without protection of all beaches, a proportion of the population is vulnerable to nest loss and mortality.

### Movements During Inter-nesting

Nest site fidelity was not correlated to in-water distance moved during the inter-nesting period. Some turtles moved little during their first inter-nesting period, but moved large distances during their second inter-nesting period (see Figure 2b, Turtle 108172). In contrast, some turtles that moved great distances during the inter-nesting period returned to nest in close proximity to their original nest site (i.e. exhibited nest site fidelity; see Figure 2b, Turtle 119940). This suggests no clear relationship between inter-nesting movements and nest-site fidelity which introduces the question: why do turtles make these long-distance movements?

In our study, 16% of individuals made long-distance (>100 km between emergences) movements during the inter-nesting period, greater than the 10% observed by Rees et al. [22] in Oman; Marcovaldi et al. [19] reported no long-distance inter-nesting movements for loggerheads in Brazil. Such long-distance movements require energy which suggests loggerheads in the northern Gulf of Mexico could be foraging during inter-nesting. Although it has been generally accepted that loggerheads do not forage during inter-nesting [81]–[83], recent studies have suggested otherwise [21], [22]. Foraging during the inter-nesting may not increase reproductive output [84] but instead may allow turtles to spread nests within the region thereby reducing the risk of complete nest loss due to a local disturbance, such as a severe erosional event [85], [33]. Whether loggerheads in the Northern Gulf subpopulation forage during inter-nesting period remains to be tested.

### Inter-nesting Area Characteristics

Because distance moved during the inter-nesting period did not correlate to nest site fidelity, turtles may have flexibility in behavior during this time. This lends further evidence to an overall plasticity in loggerhead behavior during the inter-nesting period [86], [21], [83], [22], [87]. Some turtles showed fidelity to in-water inter-nesting sites regardless of whether they remained near their original nesting site or instead made long-distance movements during the inter-nesting period. Some of these inter-nesting sites were >100 km from the original nesting site. Although loggerheads in the northern Gulf of Mexico did show variation in selection of in-water habitats, similar to results in Blumenthal et al. [21] and Rees et al. [22], a very small percentage of individuals in our study used oceanic habitat; most remained in neritic waters during the inter-nesting period.

We observed a more distinct dichotomy among individuals with respect to in-water inter-nesting habitat selection than previously surmised; some turtles showed site-fidelity to an in-water inter-nesting location whereas others were wanderers, making long-distance movements within neritic waters and showing no loyalty to a particular inter-nesting location. Although the majority of loggerheads in the northern Gulf did not leave the neritic zone (i.e., up to −200 m water depth) during inter-nesting, many made long-distance movements greater than any other loggerheads tracked to date [19], [22]. Therefore, we propose that 3 inter-nesting strategies exist for loggerheads in the northern Gulf of Mexico: 1) remain in neritic habitat near the beach where they originally nested; 2) make long-distance movements (>100 km between emergences) away from their original nesting site but remain in neritic habitat; and 3) use of oceanic habitat (in water deeper than −200 m) during the inter-nesting period (see Figure 4).

### Potential Overlap with Anthropogenic Activities

Many turtle home ranges overlapped with areas heavily used by commercial trawlers and active oil and gas developers. In their comparison of shrimping effort versus turtle density in the Gulf of Mexico, McDaniel et al. [41] previously indicated that neritic waters off of our study sites supported ‘medium’ shrimping effort but ‘low to medium’ turtle density; however, our results suggest otherwise. Many inter-nesting sites used by turtles from both study sites overlapped directly with moderate shrimping effort, as well as active oil and gas platforms (see Fig. 7). Because our SSM results indicated that neritic habitat off NW FL and AL appear to serve both as important movement corridors and inter-nesting sites for turtles nesting throughout the northern Gulf, the extent of turtle interaction with active trawling and oil and gas extraction activities may require further evaluation.

Loggerheads in near-shore northern Gulf of Mexico waters may be exposed to incidental capture in shrimp trawls, oil spills, dredging, hypoxia, and other threats. Although inter-nesting habitat characteristics and suitability for sea turtles in this region are poorly understood, locations of core-use inter-nesting habitats identified here (e.g., after the Deepwater Horizon Oil Spill) indicate that important habitat exists for loggerheads at these same potentially affected sites. Whether such at-sea inter-nesting sites previously used by loggerheads will continue to be used with equal frequency in the future, or alternatively abandoned, remains to be seen; it is possible that environmental conditions at some of these sites have been altered by the large-scale perturbation of the northern Gulf Deepwater Horizon Oil Spill [45].

### Conclusions

The results of this study highlight the vulnerability of females in this small nesting group to interactions with anthropogenic activities as well as the complexity of inter-nesting movements and habitat-use. Inter-nesting habitat-use for Northern Gulf loggerheads is not restricted to areas immediately adjacent to nesting beaches. Thus, critical habitat designations for this subpopulation, as well as subsequent management actions, should include the entire region encompassing important at-sea habitat. In addition, movement and habitat use of male loggerheads in this region is currently not well documented and future efforts are warranted to understand habitat use of both sexes; timing of male movements near breeding grounds may differ from that of females (see Hays et al. [88]. Finally, because both shrimping effort and density of petroleum extraction activities are medium to high in the northern Gulf, an increased focus on conservation actions that protect loggerheads in this subpopulation may be necessary.

## Supporting Information

Figure S1 **Examples of the predicted movement trajectory and behavioral mode for two satellite-tracked loggerhead turtles.**
(TIF)

Figure S2 **Top panel: minimum convex polygon (MCP) areas for n = 19 loggerhead turtles (** ***Caretta caretta*** **; 30 MCPs/30 centroids) tracked during inter-nesting in the Northern Gulf of Mexico during 2010 to 2012.** Tagging sites are denoted by stars, and from West to East are Gulf Shores, Alabama; Eglin Air Force Base, Florida; St. Joseph Peninsula, Florida. The box represents the extent of the bottom panel. Bottom panel: MCP centroid locations by tagging site.
(TIF)

Table S1 Observed emergence location distances for Northern Gulf loggerhead turtles (*Caretta caretta*).
(DOCX)

Table S2 Examples of posterior of switching state space model parameters for two satellite-tracked loggerhead turtles.
(DOCX)

Table S3 Northern Gulf loggerhead turtles (*Caretta caretta*) with potential inter-nesting kernel density estimates (KDEs) that failed site-fidelity tests.
(DOCX)

Table S4 Minimum convex polygon (MCP) areas for loggerhead turtle (*Caretta caretta*) inter-nesting periods with and without kernel density estimates (KDEs), with depths and distances to shore from MCP centroids.
(DOCX)

Table S5 Depths for filtered locations of satellite-tracked adult nesting loggerheads (*Caretta caretta*) in the Northern Gulf of Mexico, 2010–2012.
(DOCX)

Table S6 Shrimp trawling (data provided by NOAA) and oil and gas platform threats (from www.data.boem.gov) at or near loggerhead (*Caretta caretta*) inter-nesting minimum convex polygon (MCP) and kernel density estimate (KDE) centroids.
(DOCX)
