# Supplementary figures and images for: Movements and Habitat-Use of Loggerhead Sea Turtles in the Northern Gulf of Mexico during the Reproductive Period
Source: PLoS One. 2013 Jul 3;8(7):e66921. doi: 10.1371/journal.pone.0066921 (PMC3700946; doi:10.1371/journal.pone.0066921)

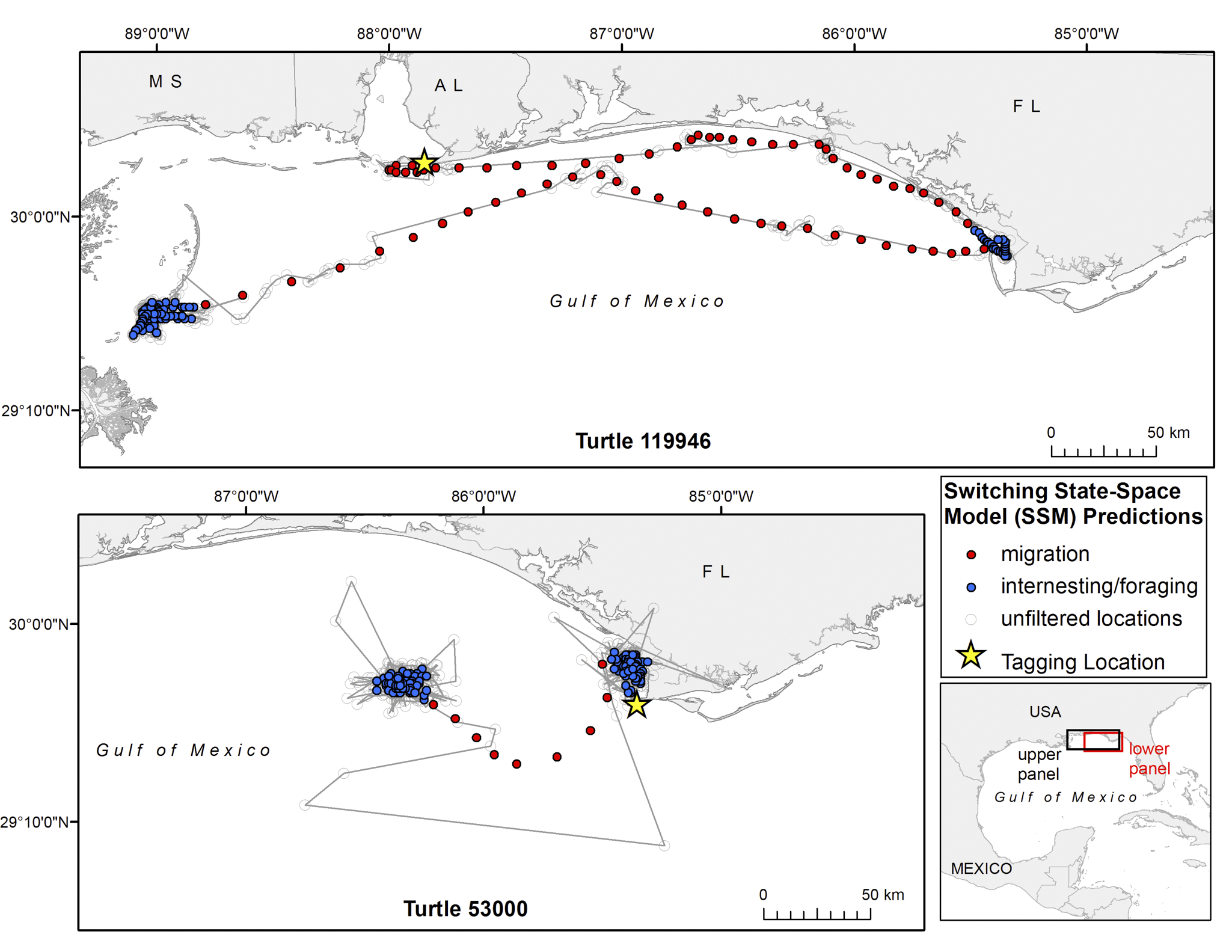

Supplement: Figure S1 — Examples of the predicted movement trajectory and behavioral mode for two satellite-tracked loggerhead turtles. (TIF) [file pone.0066921.s001.tif]

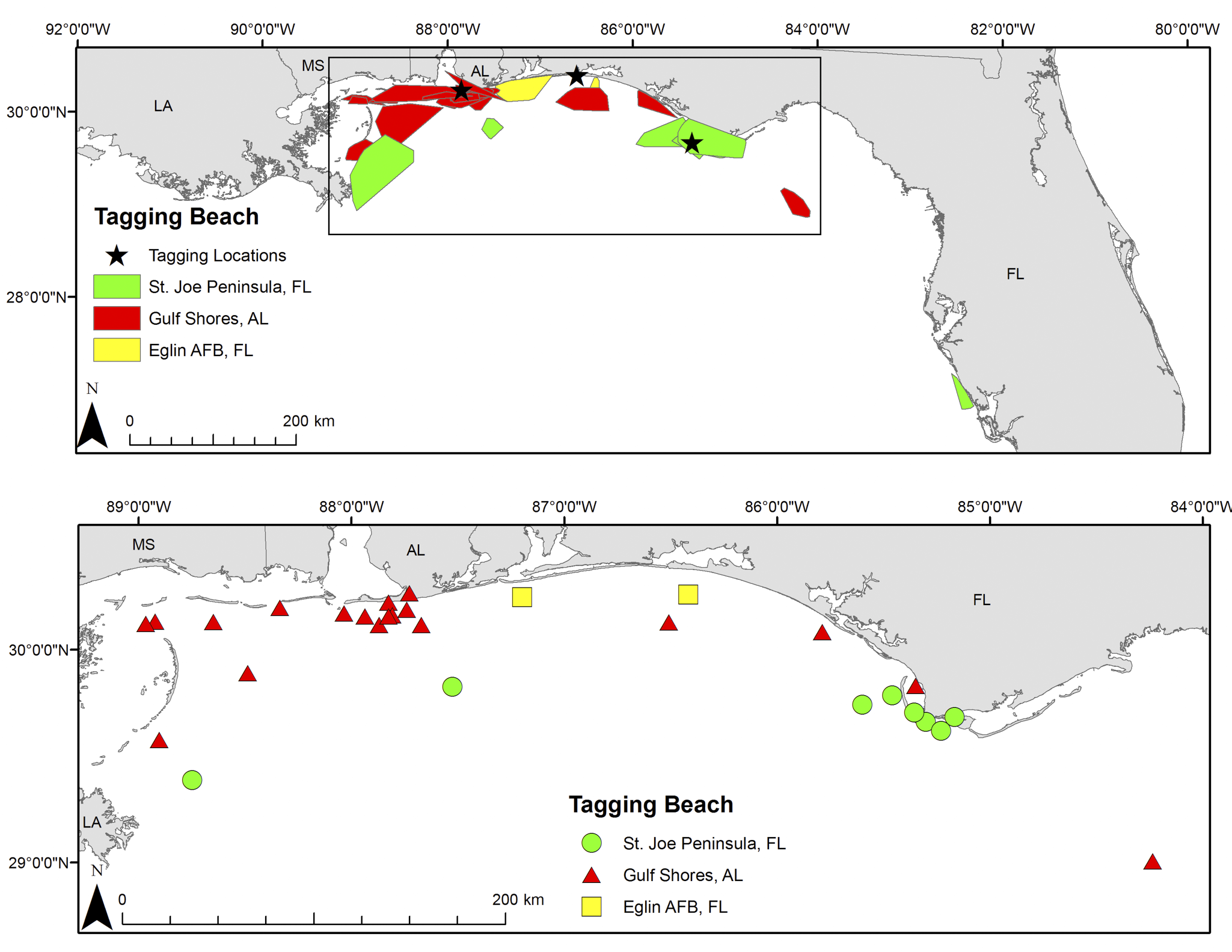

Supplement: Figure S2 — Top panel: minimum convex polygon (MCP) areas for n = 19 loggerhead turtles ( Caretta caretta ; 30 MCPs/30 centroids) tracked during inter-nesting in the Northern Gulf of Mexico during 2010 to 2012. Tagging sites are denoted by stars, and from West to East are Gulf Shores, Alabama; Eglin Air Force Base, Florida; St. Joseph Peninsula, Florida. The box represents the extent of the bottom panel. Bottom panel: MCP centroid locations by tagging site. (TIF) [file pone.0066921.s002.tif]
