# Supplementary material for: Movements and Habitat-Use of Loggerhead Sea Turtles in the Northern Gulf of Mexico during the Reproductive Period
Source: PLoS One. 2013 Jul 3;8(7):e66921. doi: 10.1371/journal.pone.0066921 (PMC3700946; doi:10.1371/journal.pone.0066921)
Supplement: Table S1 — Observed emergence location distances for Northern Gulf loggerhead turtles (Caretta caretta). (DOCX) [file pone.0066921.s003.docx]

| **Tag Number** | **Mean distance (km) between observed emergence locations (number of distances)** | **Emergence Date** | **Distance from previous emergence (km)** | **Time from previous emergence (days)** |
| --- | --- | --- | --- | --- |
| ***Gulf Shores, Alabama*** | | | | |
| 106360 | 7.1 (2) | 6/7/2011 |  |  |
|  |  | 6/22/2011 | 7.4 | 15 |
|  |  | 7/22/2011 | 6.7 | 30 |
| 108172 | 129.0 (2) | 6/8/2011 |  |  |
|  |  | 6/23/2011 | 1.6 | 15 |
|  |  | 7/22/2011 | 256.3 | 29 |
| 106345* | 3.5 (2)** | 6/9/2011 |  |  |
|  |  | 7/21/2011 | 2.7 | 42 |
|  |  | 6/7/2012 | 4.3 | NA |
| 106337 | 8.7 (2) | 6/11/2011 |  |  |
|  |  | 6/26/2011 | 12.3 | 15 |
|  |  | 7/24/2011 | 5.1 | 28 |
| 119940 | 13.0 (2) | 6/1/2012 |  |  |
|  |  | 6/17/2012 | 11.3 | 16 |
|  |  | 7/10/2012 | 14.7 | 23 |
| 119941 |  | 6/3/2012 |  |  |
|  |  | 6/22/2012 | 118.7 | 19 |
| 119924 |  | 6/6/2012 |  |  |
|  |  | 6/18/2012 | 7.8 | 12 |
| 119946 |  | 6/9/2012 |  |  |
|  |  | 6/22/2012 | 254.6 | 13 |
| 119923 | 12.9 | 6/13/2012 |  |  |
|  |  | 6/25/2012 | 12.9 | 12 |
| ***St. Joe Peninsula, Florida*** | | | | |
| 57656 |  | 7/2/2010 |  |  |
|  |  | 7/26/2010 | 1.7 | 24 |
| 89971 | 2.2 (4) | 6/1/2010 |  |  |
|  |  | 6/15/2010 | 1.7 | 14 |
|  |  | 6/28/2010 | 0.7 | 13 |
|  |  | 7/9/2010 | 1.0 | 11 |
|  |  | 7/27/2010 | 5.4 | 18 |
| 47755 | 0.2 (2) | 7/10/2010 |  |  |
|  |  | 7/22/2010 | 0.1 | 12 |
|  |  | 8/3/2010 | 0.3 | 12 |
| 53017 |  | 6/2/2012 |  |  |
|  |  | 6/28/2012 | 2.0 | 26 |
| 53016 | 4.4 (3) | 6/4/2012 |  |  |
|  |  | 6/17/2012 | 5.2 | 13 |
|  |  | 6/30/2012 | 6.3 | 13 |
|  |  | 7/13/2012 | 1.8 | 13 |
| 53164 | 2.4 (4) | 5/27/2012 |  |  |
|  |  | 6/8/2012 | 2.8 | 12 |
|  |  | 6/20/2012 | 1.2 | 12 |
|  |  | 7/2/2012 | 1.3 | 12 |
|  |  | 7/13/2012 | 4.1 | 11 |
| 119942 | 1.4 (2) | 6/10/2012 |  |  |
|  |  | 7/8/2012 | 0.3 | 28 |
|  |  | 7/22/2012 | 2.5 | 14 |
| 119950 | 3.0 (3) | 5/28/2012 |  |  |
|  |  | 6/11/2012 | 1.4 | 14 |
|  |  | 7/8/2012 | 2.2 | 27 |
|  |  | 7/19/2012 | 5.5 | 11 |
| 119949 | 1.7 (3) | 6/11/2012 |  |  |
|  |  | 6/23/2012 | 0.2 | 12 |
|  |  | 7/6/2012 | 3.3 | 13 |
|  |  | 7/20/2012 | 1.7 | 14 |
| 119948 | 1.7 (4) | 5/27/2012 |  |  |
|  |  | 6/10/2012 | 3.0 | 14 |
|  |  | 6/23/2012 | 0.3 | 13 |
|  |  | 7/5/2012 | 0.7 | 12 |
|  |  | 7/17/2012 | 2.7 | 12 |
| 119952a |  | 6/13/2012 |  |  |
|  |  | 7/6/2012 | 402.1 | 23 |
| 119952 | 1.8 (2) | 6/30/2012 |  |  |
|  |  | 7/12/2012 | 3.3 | 12 |
|  |  | 7/23/2012 | 0.4 | 11 |
| ***Eglin AFB, Florida*** | | | | |
| 120439 |  | 7/11/2012 |  |  |
|  |  | 8/2/2012 | 54.8 | 22 |
|  |  |  |  |  |
| *This turtle was tracked/observed in 2011 and 2012 | | | | |
| ** This value includes the distance between nests of 2011 and 2012. | | | | |
| NA = not available. |  |  |  |  |
