# Supplementary material for: Movements and Habitat-Use of Loggerhead Sea Turtles in the Northern Gulf of Mexico during the Reproductive Period
Source: PLoS One. 2013 Jul 3;8(7):e66921. doi: 10.1371/journal.pone.0066921 (PMC3700946; doi:10.1371/journal.pone.0066921)
Supplement: Table S2 — Examples of posterior of switching state space model parameters for two satellite-tracked loggerhead turtles. (DOCX) [file pone.0066921.s004.docx]

| **Node** | **Mean** | **SD** | **2.5% CI** | **97.5% CI** |
| --- | --- | --- | --- | --- |
| Process uncertainty from mode 1 to 1 (σ1,1) | 0.1319 | 0.0271 | 0.0874 | 0.1958 |
| Process uncertainty from mode 1 to 2 (σ1,2) | 0.0125 | 0.0251 | -0.0375 | 0.0613 |
| Process uncertainty from mode 2 to 1 (σ1,2) | 0.0125 | 0.0251 | -0.0375 | 0.0613 |
| Process uncertainty from model 2 to 2 (σ2,2) | 0.1023 | 0.0235 | 0.0643 | 0.1525 |
| Probability of being mode 1 (α1) | 0.9962 | 0.0041 | 0.9841 | 0.9999 |
| Probability of being mode 2 (α2) | 0.0955 | 0.074 | 0.0794 | 0.2928 |
| Moving speed persistence in mode 1 (γ1) | 0.4447 | 0.0977 | 0.2591 | 0.6279 |
| Moving speed persistence in mode 2 (γ2) | 0.8041 | 0.1459 | 0.4957 | 0.9938 |
