# Supplementary material for: Movements and Habitat-Use of Loggerhead Sea Turtles in the Northern Gulf of Mexico during the Reproductive Period
Source: PLoS One. 2013 Jul 3;8(7):e66921. doi: 10.1371/journal.pone.0066921 (PMC3700946; doi:10.1371/journal.pone.0066921)
Supplement: Table S3 — Northern Gulf loggerhead turtles (Caretta caretta) with potential inter-nesting kernel density estimates (KDEs) that failed site-fidelity tests. (DOCX) [file pone.0066921.s005.docx]

| **Tag Number** | **Tagging Site** | **Year** | **Inter-nesting dates used for KDE (days)** | **Site Fidelity Test** | **p value, site fidelity** |
| --- | --- | --- | --- | --- | --- |
| 119923 | AL | 2012 | 6/13/2012-7/15/2012 (32) | Fail | *p > 55.4* |
| 119942 | SJP | 2012 | 6/10/2012-7/16/2012 (36) | Fail | *p > 66.3* |
| 119950 | SJP | 2012 | 6/11/2012-8/5/2012 (55) | Fail | *p > 84.2* |
| 120439 | EAFB | 2012 | 7/12/2012-8/2/2012 (21) | Fail | *p > 88.1* |
| 53017 | SJP | 2012 | 6/4/2012-6/26/2012 (22) | Fail | *p > 75.2* |
| 119942 | SJP | 2012 | 8/5/2012-8/31/2012 (26) | Fail | *p > 86.1* |
