# Supplementary material for: Movements and Habitat-Use of Loggerhead Sea Turtles in the Northern Gulf of Mexico during the Reproductive Period
Source: PLoS One. 2013 Jul 3;8(7):e66921. doi: 10.1371/journal.pone.0066921 (PMC3700946; doi:10.1371/journal.pone.0066921)
Supplement: Table S5 — Depths for filtered locations of satellite-tracked adult nesting loggerheads (Caretta caretta) in the Northern Gulf of Mexico, 2010–2012. (DOCX) [file pone.0066921.s007.docx]

| **Tag Number** | **Mean depth (m)** | **0-50 m** | **%** | **51-100 m** | **%** | **101-150 m** | **%** | **151-200 m** | **%** | **>200 m** | **%** | **Total locations** |
| --- | --- | --- | --- | --- | --- | --- | --- | --- | --- | --- | --- | --- |
| ***Gulf Shores, Alabama*** | | | | | | | | | | | | |
| 108170 | -24.9 | 172 | 92.0 | 10 | 5.3 | 5 | 2.7 | 0 | 0.0 | 0 | 0.0 | 187 |
| 106360 | -10.5 | 130 | 100.0 | 0 | 0.0 | 0 | 0.0 | 0 | 0.0 | 0 | 0.0 | 130 |
| 108172 | -45.5 | 148 | 56.7 | 106 | 40.6 | 1 | 0.4 | 0 | 0.0 | 6 | 2.3 | 261 |
| 106345* | -21.2 | 282 | 98.9 | 3 | 1.1 | 0 | 0.0 | 0 | 0.0 | 0 | 0.0 | 285 |
| 108171 | -16.4 | 496 | 100.0 | 0 | 0.0 | 0 | 0.0 | 0 | 0.0 | 0 | 0.0 | 496 |
| 106337 | -15.8 | 136 | 97.8 | 2 | 1.4 | 0 | 0.0 | 1 | 0.7 | 0 | 0.0 | 139 |
| 108173 | -35.4 | 254 | 76.5 | 75 | 22.6 | 3 | 0.9 | 0 | 0.0 | 0 | 0.0 | 332 |
| 108174 | -24.1 | 259 | 95.6 | 4 | 1.5 | 8 | 3.0 | 0 | 0.0 | 0 | 0.0 | 271 |
| 106358 | -22.3 | 126 | 97.7 | 2 | 1.6 | 0 | 0.0 | 0 | 0.0 | 1 | 0.8 | 129 |
| 106361 | -20.0 | 201 | 100.0 | 0 | 0.0 | 0 | 0.0 | 0 | 0.0 | 0 | 0.0 | 201 |
| 108961 | -11.2 | 308 | 99.7 | 1 | 0.3 | 0 | 0.0 | 0 | 0.0 | 0 | 0.0 | 309 |
| 108964 | -56.6 | 44 | 21.7 | 153 | 75.4 | 5 | 2.5 | 1 | 0.5 | 0 | 0.0 | 203 |
| 108965 | -29.9 | 214 | 93.9 | 8 | 3.5 | 4 | 1.8 | 2 | 0.9 | 0 | 0.0 | 228 |
| 119940 | -29.3 | 347 | 88.5 | 34 | 8.7 | 9 | 2.3 | 2 | 0.5 | 0 | 0.0 | 392 |
| 119941 | -23.6 | 263 | 94.6 | 14 | 5.0 | 1 | 0.4 | 0 | 0.0 | 0 | 0.0 | 278 |
| 119943 | -25.7 | 234 | 100.0 | 0 | 0.0 | 0 | 0.0 | 0 | 0.0 | 0 | 0.0 | 234 |
| 119938 | -23.3 | 571 | 98.8 | 7 | 1.2 | 0 | 0.0 | 0 | 0.0 | 0 | 0.0 | 578 |
| 119924 | -34.6 | 525 | 83.7 | 91 | 14.5 | 8 | 1.3 | 1 | 0.2 | 2 | 0.3 | 627 |
| 119944* | -30.7 | 249 | 99.2 | 1 | 0.4 | 0 | 0.0 | 1 | 0.4 | 0 | 0.0 | 251 |
| 119946 | -7.8 | 322 | 98.5 | 5 | 1.5 | 0 | 0.0 | 0 | 0.0 | 0 | 0.0 | 327 |
| 119945 | -531.2 | 66 | 49.3 | 25 | 18.7 | 1 | 0.7 | 2 | 1.5 | 40 | 29.9 | 134 |
| 119947 | -14.5 | 219 | 100.0 | 0 | 0.0 | 0 | 0.0 | 0 | 0.0 | 0 | 0.0 | 219 |
| 119923 | -27.8 | 507 | 99.2 | 4 | 0.8 | 0 | 0.0 | 0 | 0.0 | 0 | 0.0 | 511 |
| ***St. Joe Peninsula, Florida*** | | | | | | | | | | | | |
| 57656 | -758.3 | 72 | 55.0 | 8 | 6.1 | 3 | 2.3 | 6 | 4.6 | 42 | 32.1 | 131 |
| 89971 | -31.0 | 136 | 98.6 | 2 | 1.4 | 0 | 0.0 | 0 | 0.0 | 0 | 0.0 | 138 |
| 47755 | -1143.1 | 21 | 43.8 | 3 | 6.3 | 2 | 4.2 | 0 | 0.0 | 22 | 45.8 | 48 |
| 52968 | -20.4 | 80 | 100.0 | 0 | 0.0 | 0 | 0.0 | 0 | 0.0 | 0 | 0.0 | 80 |
| 53017 | -7.6 | 91 | 100.0 | 0 | 0.0 | 0 | 0.0 | 0 | 0.0 | 0 | 0.0 | 91 |
| 53016 | -8.9 | 117 | 100.0 | 0 | 0.0 | 0 | 0.0 | 0 | 0.0 | 0 | 0.0 | 117 |
| 53000 | -42.5 | 211 | 59.9 | 96 | 27.3 | 44 | 12.5 | 1 | 0.3 | 0 | 0.0 | 352 |
| 53164 | -7.0 | 75 | 100.0 | 0 | 0.0 | 0 | 0.0 | 0 | 0.0 | 0 | 0.0 | 75 |
| 119942 | -19.2 | 547 | 96.1 | 21 | 3.7 | 1 | 0.2 | 0 | 0.0 | 0 | 0.0 | 569 |
| 119950 | -18.4 | 128 | 100.0 | 0 | 0.0 | 0 | 0.0 | 0 | 0.0 | 0 | 0.0 | 128 |
| 119949 | -9.2 | 126 | 100.0 | 0 | 0.0 | 0 | 0.0 | 0 | 0.0 | 0 | 0.0 | 126 |
| 119951 | -8.6 | 118 | 100.0 | 0 | 0.0 | 0 | 0.0 | 0 | 0.0 | 0 | 0.0 | 118 |
| 119948 | -9.5 | 240 | 100.0 | 0 | 0.0 | 0 | 0.0 | 0 | 0.0 | 0 | 0.0 | 240 |
| 119952a | -15.4 | 70 | 100.0 | 0 | 0.0 | 0 | 0.0 | 0 | 0.0 | 0 | 0.0 | 70 |
| 119952 | -35.4 | 59 | 98.3 | 0 | 0.0 | 0 | 0.0 | 0 | 0.0 | 1 | 1.7 | 60 |
| ***Eglin AFB, Florida*** | | | | | | | | | | | | |
| 120438 | -408.4 | 228 | 70.8 | 41 | 12.7 | 3 | 0.9 | 2 | 0.6 | 48 | 14.9 | 322 |
| 120439 | -18.0 | 149 | 100.0 | 0 | 0.0 | 0 | 0.0 | 0 | 0.0 | 0 | 0.0 | 149 |
| ***TOTAL*** |  | *8541* |  | *716* |  | *98* |  | *19* |  | *162* |  | *9536* |
| ***%*** |  | *89.6* |  | *7.5* |  | *1.0* |  | *0.2* |  | *1.7* |  |  |
|  |  |  |  |  |  |  |  |  |  |  |  |  |
| *Same turtle tracked/observed in 2011 and 2012. | | | | | | | | | | | | |
