# Supplementary material for: Movements and Habitat-Use of Loggerhead Sea Turtles in the Northern Gulf of Mexico during the Reproductive Period
Source: PLoS One. 2013 Jul 3;8(7):e66921. doi: 10.1371/journal.pone.0066921 (PMC3700946; doi:10.1371/journal.pone.0066921)
Supplement: Table S6 — Shrimp trawling (data provided by NOAA) and oil and gas platform threats (from www.data.boem.gov) at or near loggerhead (Caretta caretta) inter-nesting minimum convex polygon (MCP) and kernel density estimate (KDE) centroids. (DOCX) [file pone.0066921.s008.docx]

|  | **Centroid 1** | | | **Centroid 2** | | | **Centroid 3** | | | **Centroid 4** | | |
| --- | --- | --- | --- | --- | --- | --- | --- | --- | --- | --- | --- | --- |
| **Tag Number** | **Trawling Days** | **Trawling level** | **Platforms** | **Trawling Days** | **Trawling level** | **Platforms** | **Trawling Days** | **Trawling level** | **Platforms** | **Trawling Days** | **Trawling level** | **Platforms** |
| ***Gulf Shores, Alabama*** | | | | | | | | | | | | |
| 106360 | 1778.8 | 3 | 3 |  |  |  |  |  |  |  |  |  |
| 108172 | 1778.8 | 3 | 3 | 3.8 | 1 | 0 |  |  |  |  |  |  |
| 106345* | 1778.8 | 3 | 6 | 1778.8 | 3 | 0 | 2082.5 | 3 | 0 |  |  |  |
| 106337 | 4543.4 | 4 | 0 | 1778.8 | 3 | 8 | 1778.8 | 3 | 12 | 2082.5 | 3 | 0 |
| 119940 | 1778.8 | 3 | 3 |  |  |  |  |  |  |  |  |  |
| 119941 | 4543.4 | 4 | 0 |  |  |  |  |  |  |  |  |  |
| 119943 | 819.5 | 2 | 5 | 1778.8 | 3 | 1 | 2082.5 | 3 | 0 |  |  |  |
| 119924 | 1778.8 | 3 | 13 | 1778.8 | 3 | 15 | 819.5 | 2 | 0 |  |  |  |
| 119944* | 4543.4 | 4 | 0 | 2082.5 | 3 | 0 |  |  |  |  |  |  |
| 119946 | 2095.7 | 3 | 0 | 1778.8 | 3 | 26 |  |  |  |  |  |  |
| 119947 | 1778.8 | 3 | 13 |  |  |  |  |  |  |  |  |  |
| 119923 | 1778.8 | 3 | 7 | 2082.5 | 3 | 0 |  |  |  |  |  |  |
| ***St. Joe Peninsula, Florida*** | | | | | | | | | | | | |
| 53017 | 424.6 | 2 | 0 |  |  |  |  |  |  |  |  |  |
| 53016 | 424.6 | 2 | 0 |  |  |  |  |  |  |  |  |  |
| 53000 | 2095.7 | 3 | 0 | 3.8 | 1 | 0 |  |  |  |  |  |  |
| 53164 | 424.6 | 2 | 0 |  |  |  |  |  |  |  |  |  |
| 119942 | 2095.7 | 3 | 0 | 819.5 | 2 | 6 |  |  |  |  |  |  |
| 119950 | 2082.5 | 3 | 0 |  |  |  |  |  |  |  |  |  |
| 119949 | 424.6 | 2 | 0 |  |  |  |  |  |  |  |  |  |
| 119951 | 2095.7 | 3 | 0 |  |  |  |  |  |  |  |  |  |
| 119952a | 424.6 | 2 | 0 |  |  |  |  |  |  |  |  |  |
| 119952 | 424.6 | 2 | 0 | 819.5 | 2 | 0 |  |  |  |  |  |  |
| ***Eglin AFB, Florida*** | | | | | | | | | | | | |
| 120438 | 2082.5 | 3 | 0 |  |  |  |  |  |  |  |  |  |
| 120439 | 819.5 | 2 | 0 |  |  |  |  |  |  |  |  |  |
|  |  |  |  |  |  |  |  |  |  |  |  |  |
| *Same turtle tracked/observed in 2011 and 2012. | | | | | | | | | | | | |
