# Supplementary material for: Movements and Habitat-Use of Loggerhead Sea Turtles in the Northern Gulf of Mexico during the Reproductive Period
Source: PLoS One. 2013 Jul 3;8(7):e66921. doi: 10.1371/journal.pone.0066921 (PMC3700946; doi:10.1371/journal.pone.0066921)
Supplement: Supplementary file 7 [file pone.0066921.s006.docx]

			MCP results[a]															
Tag Number	Average MCP centroid distance to shore (km)	Average MCP centroid depth (m)	Date Range	Area (km^2)	Centroid distance (km)	Centroid depth (m)	Date Range	Area (km^2)	Centroid distance (km)	Centroid depth (m)	Date Range	Area (km^2)	Centroid distance (km)	Centroid depth (m)	Date Range	Area (km^2)	Centroid distance (km)	Centroid depth (m)
106360			*6/7-8/4/2011*	*966.1*	*KDE*	*KDE*												
108172	5.8	-12.0	6/8-7/1/2011	473.8	5.8	-12.0	*8/11-8/31/2011*	*1284.5*	*KDE*	*KDE*								
106345	14.6	-20.0	6/9-7/3/2011	1408.8	3.5	-8.0	7/10-7/17/2011	204.6	11.1	-15.0	7/28-8/31/2011	1189.9	29.1	-37.0				
106337	5.1	-10.3	6/11-6/19/2011	374.0	0.0	0.0	6/24-7/12/2011	247.9	5.8	-7.0	7/15-8/7/2011	303.5	8.1	-13.0	8/14-8/31/2011	490.5	6.4	-21.0
119940	0.7	-9.0	7/11-8/7/2012	202.9	0.7	-9.0												
119941	9.9	-9.0	7/13-7/26/2012	150.6	9.9	-9.0												
119943	46.6	-25.0	6/12-6/25/2012	2165.8	30.8	-30.0	6/30-7/4/2012	388.1	15.1	-16.0	8/2-8/29/2012	557.1	94.0	-29.0				
119924	10.7	-14.5	6/6-6/22/2012	568.7	8.5	-13.0	7/2-7/4/2012	24.5	12.9	-17.0	*7/16-8/31/2012*	*1499.8*	*KDE*	*KDE*				
119944	9.3	-10.0	6/10-6/17/2012	258.5	9.3	-10.0	*6/28-8/29/2012*	*809.1*	*KDE*	*KDE*								
119946	6.0	-8.0	6/23-7/5/2012	3.0	2.2	-9.0	7/17-8/29/2012	672.2	9.8	-7.0								
119947			*6/13-8/12/2012*	*1058.0*	*KDE*	*KDE*												
119923	7.6	-14.0	6/13-7/15/2012	1016.4	7.6	-14.0	*7/31-8/31/2012*	*1134.9*	*KDE*	*KDE*								
53017	2.7	-7.0	6/4-6/26/2012	571.0	2.7	-7.0												
53016			*6/5-7/17/2012*	*765.4*	*KDE*	*KDE*												
53000			*6/8-7/24/2012*	*356.0*	*KDE*	*KDE*	*7/28-8/27/2012*	*902.8*	*KDE*	*KDE*								
53164			*6/8-7/14/2012*	*195.9*	*KDE*	*KDE*												
119942	16.2	-21.5	6/10-7/16/2012	2218.4	0.3	0.0	8/5-8/31/2012	3274.2	32.2	-43.0								
119950	18.1	-26.0	6/11-8/5/2012	1439.3	18.1	-26.0												
119949	6.5	-11.0	6/11-7/12/2012	940.7	6.5	-11.0												
119951	1.5	-1.0	6/11-7/18/2012	951.9	1.5	-1.0												
119952a	5.5	-10.0	6/29-7/6/2012	380.0	5.5	-10.0												
119952	27.4	-24.5	7/23-8/4/2012	79.4	4.3	-11.0	8/15-8/29/2012	329.0	50.5	-38.0								
120438	13.7	-27.0	7/22-7/25/2012	174.0	13.7	-27.0												
120439	8.4	-23.0	7/12-8/2/2012	1182.1	8.4	-23.0												

[a]Italics also have a kernel density estimation for the same date range.
